# Colonization history and population differentiation of the Honey Bees (*Apis mellifera* L.) in Puerto Rico

**DOI:** 10.1002/ece3.5330

**Published:** 2019-09-28

**Authors:** Jenny P. Acevedo‐Gonzalez, Alberto Galindo‐Cardona, Arian Avalos, Charles W. Whitfield, Dania M. Rodriguez, Jose L. Uribe‐Rubio, Tugrul Giray

**Affiliations:** ^1^ Department of Biology University of Puerto Rico San Juan Puerto Rico; ^2^ National Scientific and Technical Research Council (CONICET) Tucuman Argentina; ^3^ Miguel Lillo Foundation Tucumán Argentina; ^4^ USDA, Agricultural Research Service Honey Bee Breeding, Genetics and Physiology Research Baton Rouge Louisiana; ^5^ Department of Entomology University of Illinois at Urbana‐Champaign Urbana Illinois; ^6^ CENIDFA‐INIFAP Animal Physiology Research Center Ajuchitlán Mexico

**Keywords:** Africanized honey bees, gentle behavior, hybrid population, SNPs

## Abstract

Honey bees (*Apis mellifera* L.) are the primary commercial pollinators across the world. The subspecies *A. m. scutellata* originated in Africa and was introduced to the Americas in 1956. For the last 60 years, it hybridized successfully with European subspecies, previous residents in the area. The result of this hybridization was called Africanized honey bee (AHB). AHB has spread since then, arriving to Puerto Rico (PR) in 1994. The honey bee population on the island acquired a mosaic of features from AHB or the European honey bee (EHB). AHB in Puerto Rico shows a major distinctive characteristic, docile behavior, and is called gentle Africanized honey bees (gAHB). We used 917 SNPs to examine the population structure, genetic differentiation, origin, and history of range expansion and colonization of gAHB in PR. We compared gAHB to populations that span the current distribution of *A. mellifera* worldwide. The gAHB population is shown to be a single population that differs genetically from the examined populations of AHB. Texas and PR groups are the closest genetically. Our results support the hypothesis that the Texas AHB population is the source of gAHB in Puerto Rico.

## Graphical abstract

AHB on PR hybridized with EHB and processes of local selection and extraordinary features of the island resulted in an “island bee” currently called gAHB. The ancestral parental gAHB came from Texas. The gAHB population have diverged from its origin (Texas) and is a population with a distinct stable genetic structure. Our results suggest that gAHB may represent a new ecotype of Apis mellifera.

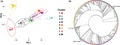

## 1 INTRODUCTION

The gentle Africanized honey bees of Puerto Rico (gAHB) are a unique population that combines some desirable traits, such as mite resistance (intense grooming and biting behavior that does not allow the proliferation of the mites). These bees have not been affected by losses common in the US and the world, as population samples showed an absence or low levels of seven viruses monitored in the National Honey Bee Health Survey (Madella et al., 2016). Also, gAHB have reduced colony defensiveness (Rivera‐Marchand, Oskay and Giray, 2012), and the least defensive colonies show the highest rate of foraging and honey reserves (Rivera‐Marchand, Giray, & Guzmán‐Novoa, 2008). This admixed population is part of the broader history of the accidental introduction of Africanized honey bees (AHB) to continental Brazil and later spread across the Neotropics and southern Nearctic. Since its introduction and spread, AHB has had significant ecological, agricultural, and human impact (Morse et al., 1973; Sheppard et al., 1991; Sheppard et al., 1991; Nelson et al., 2017). As part of this expansion and assisted by human transit, AHB arrived to Puerto Rico in 1994 (Cox, 1994). However, AHB's continental origin remains unknown and only one introduction event is thought to have occurred (see Rivera‐Marchand et al., 2008; Galindo‐Cardona et al., 2013).

Like the rest of the New World, Puerto Rico had an existing population of EHB, which were introduced by colonizers (Engel, 1999; Horn, 2005) prior to the arrival of AHB on the island. These EHB were likely an admixed population combining genetic diversity from current commercial “Italian” strains (C group) and initial historical stocks from Spain (M group) (Phillips, 1914; Taylor, 1977; Whitfield et al., 2006). However, by the time AHB arrived, this initial EHB population had been severely negatively impacted by the 1980s introduction of *Varroa* (de Guzman, Rinderer and Stelzer, 1997). Mirroring continental patterns, the introduced AHB hybridized and broadly displaced the already battered EHB population. In contrast with other continental AHB populations, Puerto Rico's remoteness has since limited continued AHB gene flow.

Isolation and other factors unique to Puerto Rico as a densely populated oceanic island have resulted in the unique characteristics that distinguish AHB there. For instance, gAHB are gentle in levels comparable to managed EHB colonies (Rivera‐Marchand et al., 2008; Rivera‐Marchand, Oskay and Giray, 2012) yet they are resistant to the *Varroa* mite, which is a vector for various viruses (Guzman‐Novoa & Correa‐Benitez, 1996). In addition, honey bee colonies in Puerto Rico have not been affected by the degree of losses common in mainland US and other parts of the world (e.g., Oldroyd, 2007; Giray et al., 2010).

Though much is known about the events surrounding introduction and spread of AHB in the island and the selective pressures it experienced to become the gAHB (Avalos et al., 2017), the genetic origin and patterns of admixture of this population remain poorly understood. Past studies identified that gAHB is a contiguous population spanning Puerto Rico and two adjacent islands (Vieques, Culebra) with no detectable population substructure (Galindo‐Cardona et al., 2013). Analysis of parental lineage through mitotype identification showed a single African matriline present in the island, in contrast with five detected in continental AHB populations (Rivera‐Marchand et al., 2008). In addition, we know the population has retained a sizeable proportion of EHB alleles, with a suggested 40% introgression (Galindo‐Cardona et al., 2013). Identifying the putative AHB founding population giving rise to gAHB can help understand the range and changes in genetic diversity leading to the evolution of this unique population and further inform how allelic profiles conferring both reduced colony defensiveness and parasite resistance may arise (Hunt et al., 2007; Navajas et al., 2008; Tsuruda et al., 2012).

In this study, we capitalize on a previous data set representing the widest geographical sampling available to date for honey bees (Whitfield et al., 2006), albeit with a greater representation of Africanized honey bees. We expand on this coverage by adding samples from the gAHB population in Puerto Rico. We implemented the combined data set to elucidate the recent genetic history of gAHB. Specifically, we address three major aims: (a) to describe the genetic structure and ancestry contributions to the gAHB population in Puerto Rico, (b) to assess the geographic origin of gAHB parental populations, and (c) to examine the possible existence of populations with similar genetic profiles to that of gAHB in the broader spectrum of continental AHB genetic diversity. In addition, we assess if gAHB were a genetic mosaic in parts of its genome by contrasting whether alleles from one of the parental lineages were more frequent in gAHB than expected for particular markers. These aims provide a critical biogeographical context for a population known for its evolutionary novelty, furthering projects on current and future traits of interest.

## 2 MATERIALS AND METHODS

### 2.1 Data collection and processing

A total of 40 gAHB samples were collected from the Gurabo Apiary in Puerto Rico (18°15′27.65″N, 65°59′11.16″W, Figure 1 in Galindo‐Cardona et al., 2013). To prevent oversampling of maternal alleles, only one bee per colony was subjected to genetic analysis. Samples were of different pupal stages to ensure colony origin. Genomic DNA from half the thorax of an individual honey bee was extracted using DNeasy extraction kit from QIAGEN^®^ with the animal tissue protocol. The extracted DNA was assessed using agarose gel electrophoresis (1%), NanoDrop (NanoDrop ND‐1000), and Qubit Fluorometer (Invitrogen™), according to the manufacturer's instructions.

**Figure 1:**
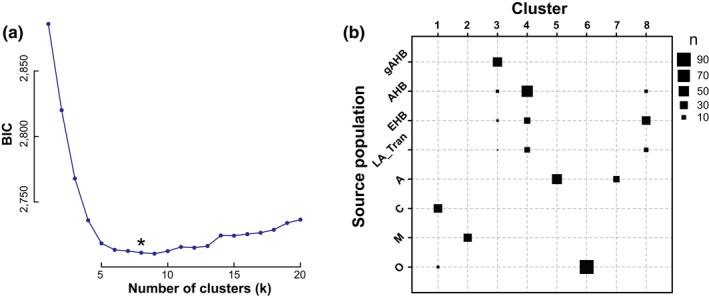
Identification of unsupervised genetic clustering via k‐means selection. a, Plot of the Bayesian information criteria (y‐axis) used to select the optimal number of possible genetic clusters (x‐axis) in our data set. A k = 8 number of clusters was optimal for this data set (highlighted by an asterisk). b, The plot illustrates relationship of cluster memberships between prior population clusters (y‐axis) and derived unsupervised genetic clusters (x‐axis) for the data set. Square size indicates number of samples as defined in the legend

### 2.2 Genotyping

The data were obtained with the same SNP panel used by Whitfield and colleagues (Whitfield et al., 2006). Briefly, we used Illumina's Bead Array Technology and the Illumina GoldenGate^®^ allele‐specific extension assay (Illumina) with a custom Oligo Pool Assay (OPA), following manufacturer's protocols. Activated DNA targets were bound with allele‐specific oligo (ASO), each dyed differently at the imaging stage (Whitfield et al., 2006).

### 2.3 Reference data set

Our genotyping approach identified 1,136 SNPs for the 40 gAHB samples. We combined these with the data set provided in Whitfield et al. (2006) which includes genotypes of 330 individuals from 8 major genetic groups including 14 subspecies and geographic and temporal transects for three other populations. Joining the two sets, we arrived at 917 SNPs after identifying concordant marker locations and removing markers that were monomorphic across the data set or poorly represented (only 2% of the samples across the populations). We also established a priori bins for the samples using geographic locations or parentage determination when available. This resulted in 8 distinct sample groups with four corresponding to known ancestral lineages (C, M, O, and A groups) for honey bee, and four encompassing samples from the Western Hemisphere (gAHB, AHB, EHB, and Latin American Transect). The AHB and EHB groups were defined by mitotype information available for the samples and as reported in Whitfield et al. (2006). These two clusters contained samples from Brazil, Texas, and Arizona, and from the temporal transect quantifying Africanization in the Welder Wildlife Refuge (WWR).

### 2.4 Genetic structure and ancestry in gAHB

**Figure 2:**
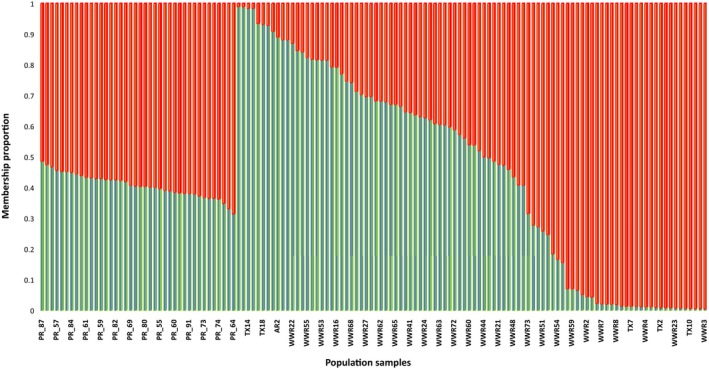
The plot shows STRUCTURE analysis, using genetic distances among the groups of honey bees from Puerto Rico (PR_) were separated from those of Texas (TX_) and World Wide Refuge (WWR_)

**Figure 3:**
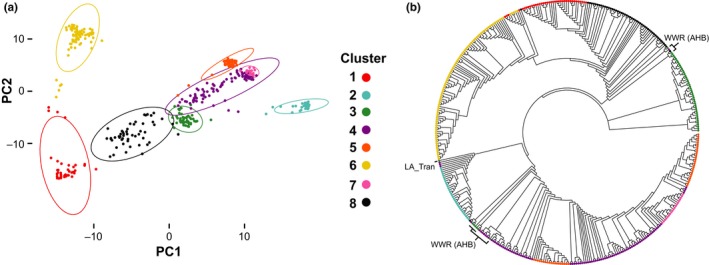
Structure clusters derived from genetic similarity across the data set. a, Principal components analysis (PCA) of the data set. b, Neighbor‐joining tree plotted as an unrooted cladogram of the same data set used to explore genetic relationship between samples and its correlate with cluster assignation. Specific labels are provided for a subset of nine samples from continental hybrid populations (WWR, Latin American Transect) grouped in the same cluster as all the gAHB samples. In both panels colors are provided to highlight the previously defined genetic clusters (as in Methods 2.4, also in Figure 1b)

We examined genetic clustering and population structure via discriminant analysis of principal components (DAPC; Jombart, Devillard, & Balloux, 2010) and STRUCTURE (Pritchard, Stephens, & Donnelly, 2000). Genetic structure via DAPC comprised the determination of optimal clusters achieved by using the *find.clusters*() function in the *adegnet* R package (Jombart et al., 2010). The approach applies successive k‐means clustering of a PCA derived from the genotype matrix (917 SNP × 370 samples) and produces a goodness of fit BIC criteria for each level of k (Figure 1a). In this analysis, k represents a “preselected parameter corresponding to an a priori number of populations or genetic groups, represented by a set of allele frequencies described in the data” (Pritchard et al., 2000). The optimal number of k and corresponding sample assignations to these clusters are used to identify the principal components that maximize differentiation between clusters while minimizing differentiation within clusters (Jombart et al., 2010). These were juxtaposed with our a priori bins of samples to outline genetic history *vis‐a‐vis* geographic distribution and parental origin (Figure 1b). A separate STRUCTURE analysis was run with gAHB (*n* = 40) and Texas AHB (*n* = 101) populations to determine differentiation of these two populations (Figure 2). We also compared DAPC cluster assignation with phylogenetic relationships between samples. Our approach used functions from the *ape* package in R to derive Euclidean distances between samples using the genotype matrix to create a per‐sample neighbor‐joining tree (Figure 3b). This way, we could examine the genetic proximity of mis‐assigned samples.

**Figure 4:**
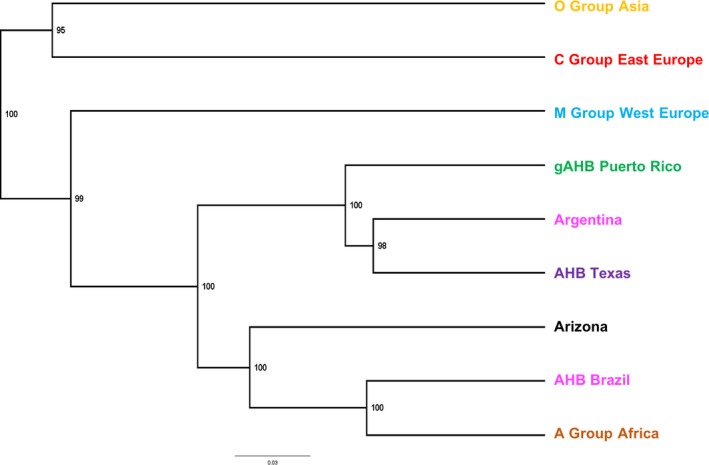
UPGMA Dendrogram. Tree based on genetic distances of Nei (1987) for different populations of *Apis mellifera* analyzed in the world, including gAHB. Colors are provided for visual representation and correspond to the cluster (Methods 2.4, Figure 1 & 3) where the majority of samples from each of the populations was assigned

### 2.5 Geographic origin of gAHB

Using the combined 370 sample × 917 SNP data set, we applied a phylogenetic analysis to identify the genetic and geographic source of gAHB. Our approach used Prevosti's absolute genetic distance (Prevosti, Ocaña, & Alonso, 1975) to quantify individual relationships within and among populations. These distance matrices were reduced to (a) a rooted dendrogram at the population level using an Unweighted Pair Group Method with Arithmetic Mean (UPGMA) hierarchical clustering strategy (Sokal and Michener, 1958), and (b) an unrooted neighbor‐joining (NJ) phylogenetic tree (Nei & Saitou, 1987). These analyses were performed using the *R* Statistical Software Language (version 3.3.2; (R Core Team, 2016)) and used the *poppr* package (version 2.4.1; Kamvar, Tabima and Grünwald, 2014; Kamvar, Brooks, & Grünwald, 2015). Specific R scripts and detailed package references are available as supplemental material (see DRYAD) (SM1).

### 2.6 Mosaic test

We tested the deviation from an admixture model for specific markers by comparing all SNP marker allele frequencies across an expected hybrid frequency of AHB (from Arizona and Texas samples) and EHB bees (Texas and Managed colonies) in the sample to gAHB allele frequencies (SAS Institute Inc., 2019). Significant deviation in allele frequencies from the expected hybrid frequency indicated either more AHB‐like or more EHB‐like loci. The number of loci with significant deviation was compared to expected by chance.

## 3 RESULTS

### 3.1 Genetic structure and ancestry in gAHB

Results of the DAPC cluster assessment identified an optimal number of K = 8 genetic clusters in the data set (Figure 1a). Clusters largely agreed with a priori bins (Figure 1b). Comparison with a priori bins also revealed a large degree of genetic overlap and variation between the gAHB, AHB, and EHB samples (Figure 1b). This variation stems from historical and current factors impacting gene flow in these populations. Specifically, gAHB, AHB, and EHB are admixed combinations of the ancestral genetic groups (A, M, O, C) derived from introgression (AHB, gAHB) and human intervention (EHB) (Kerr, 1967; Beye et al., 2006; Whitfield et al., 2006; Rivera‐Marchand, Oskay and Giray, 2012). In the case of EHB and AHB, extensive gene flow is known to happen between adjacent continental populations.

To determine if gAHB is a distinct island from other populations sampled from the range of AHB (i.e., Brazil, Argentina, Texas, see SM1 for sample identification), we set up an analysis of structure on gAHB (*n* = 40) using Texas bees (*n* = 101) (Figure 2). This analysis shows two clusters (K = 2) with similar membership proportion for all individuals of gAHB population (40). Texas, Arizona, and WWR individuals show the same clusters (2), but the membership proportion is unequal with some individuals showing membership equal to 1 (*i.e.,* Tx14 belong to one of two populations). These results indicate that some individuals from the Texas population are more similar to ancestral cluster 1 (Europe), and other individuals from the Texas population are more similar to ancestral cluster 2 (Africa). Although the Puerto Rico honey bee is also within the hybrid spectrum, it is a stable population found on the island, supporting a single, undivided population.

### 3.2 Geographic origin of gAHB

We examined the combined 917 SNP × 370 sample data set to explore the origin of gAHB in Puerto Rico. We included New World groups (Texas, Brazil and gAHB) into the STRUCTURE analysis using genetic distances among the groups (Figure 4). The analysis shows groups in three nodes: (a) the populations of Asia and Eastern Europe, (b) the populations of Texas, Puerto Rico, and Argentina, and (c) the populations of Brazil, Africa, and Arizona. This analysis indicated that the population of gAHB bees was in the same node as the population of Texas. This supports one of the two hypotheses, namely a Texas origin for Puerto Rico gAHB instead of a Brazil origin.

### 3.3 Cluster assignment population

A PCA of the data set was conducted to examine population structure using the K = 8 clusters (Figure 3a). This analysis showed the gAHB cluster (Cluster 3, green) to be intermediate between the mostly AHB cluster (Cluster 4, pink) and the mostly EHB cluster (Cluster 8, orange; Figure 3a). Cluster assignation also identified some samples clustering with gAHB. Further examination revealed these samples to be from the WWR temporal transect and the Latin American geographic transect conducted by Whitfield et al., (2006). Most of the samples clustered with gAHB had African mitotypes as reported by Whitfield et al. (2006). Cross‐referencing collection dates with the identified WWR samples showed that most of the samples that fell within Cluster 3 corresponded to the early portion of the time series (1995–1996) which correlated with the earliest description of AHB in Puerto Rico (Cox, 1994). The other misidentified sample belonging to the Latin American transect laid near the AHB border of the 2006 hybrid zone by the town of Ayui, Entre Rios, Argentina, near the border with Uruguay (latitude: −31.08321667, longitude: −58.06596667).

Cluster analyses suggest that the genetic profile of gAHB lies within the spectrum of AHB‐EHB hybridization. This is further supported by the observation that samples from the transect within the Argentinian 2006 hybrid zone, though a continent apart, are genetically similar to gAHB samples. In addition, there is contribution of the M group (Cluster 2 Figure 3a) evident, concordant with historical precedence (Agra et al., 2018).

### 3.4 Mosaic test

**Figure 5:**
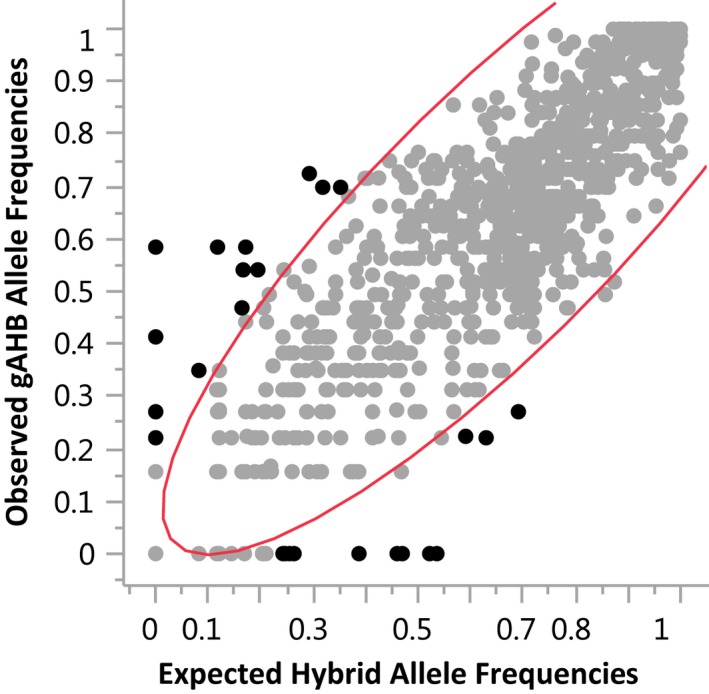
Scatterplot matrix showing the correlation coefficients, comparing all SNPs markers allele frequencies across a calculated hybrid frequency of AHB (from Arizona and Texas samples) and EHB bees (Texas and Managed colonies) in the sample of gAHB allele frequencies

We tested the mosaic hypothesis by comparing all SNP marker allele frequencies across a calculated hybrid frequency of AHB (from Arizona and Texas samples) and EHB bees (Texas and Managed colonies) in the samples of gAHB allele frequencies. The correlation coefficient for this comparison was 0.86 (*r* = 0.86, *df* = 916, *p* < 0.01), and Mahalanobis distance analysis revealed 60 SNPs that were outliers; they had allele frequencies with a significant deviation from the expected admixture frequency (Figure 5). This is greater than 6.5% of all SNPs, indicating that there are at least six times more loci than what either parental population would resemble by chance (cut off for outliers was <0.01), supporting the mosaic hypothesis. Because of markers' dispersion (*i.e.,* only ~917 total number of markers were considered, 60 outliers identified as significantly different from hybrid), associations with known genes and traits were not explored.

## 4 DISCUSSION

The genetic structure of gAHB found in Puerto Rico supports a single colonization event, as indicated by monophyly of this group. Specific phylogeographic relations indicate the potential source population to be the Texas AHB. The single colonization hypothesis was previously suggested by the presence of a single mtDNA haplotype in Puerto Rico of five available within AHB populations (Rivera‐Marchand et al., 2008). A single, uniform population was also indicated based on microsatellite markers in the study by Galindo‐Cardona and colleagues (2013). The Texas AHB origin hypothesis was also suggested based on similarity of microsatellite genetic profiles across Texas AHB and PR gAHB populations (Galindo‐Cardona et al., 2013). Our results suggest a hypothesis that the higher genetic diversity in the present population of gAHB may have allowed them to respond and adjust more efficiently to environmental changes than the EHB that preceded them in Puerto Rico (Delgado et al., 2012; Rivera‐Marchand, Oskay and Giray, 2012). gAHB is a distinct population derived from the broader AHB hybridization spectrum, further evolved on the island (Avalos et al., (2017) and Figure 3b). It appears that the colonization event c.a. 1994 (Cox, 1994) initiated a process of hybridization that after 20 years leads to the establishment of an admixed island population (Avalos et al., 2017). Currently, the island population is an important reservoir of genetic diversity with traits of high interest for apiculture and agriculture as discussed in relation to *Varroa* resistance, reduced aggressiveness, and low viral load.

One hypothesis addressing the distinct genetic variation in the gAHB population is that introgression of alleles varied in different proportion from locus to locus, making some traits mostly African, others European. Honey bees have a relatively small genome (Honey bee Genome Sequencing Consortium, 2006) and the highest recombination rate reported of any multicellular organism so far (Beye et al., 2006). These characteristics foster the rapid development of novel combinations of genetic variation. In Puerto Rico, gAHB has undergone a soft selective sweep favoring retention of genetic variation in the frequency profile of many alleles across the genome (Avalos et al., 2017) and leading to a genetic mosaic. Previously, even with only a few markers it was observed that two of eight microsatellite loci tested deviated from the expected allele frequencies based on an admixture model (Galindo‐Cardona et al., 2013). We tested this hybrid mosaic hypothesis now with 917 markers across the genome and found 6.5% of the markers to deviate from the admixture model, demonstrating the “mosaic” characteristic.

Cluster and assignment analyses converge in that (a) gAHB was most likely derived from precursors that were part of the early hybridizing population present in Texas during 1993–2000, and (b) gAHB‐like genotypes may be more common than expected and may emerge early in the AHB‐EHB admixture process (as in the continuous hybrid zone in Argentina). All the gAHB samples spring from a monophyly, while samples in the C (Cluster 1)—EHB (Cluster 8)—AHB (Cluster 4)—A (Cluster 5) spectrum span the phylogeny between these groups (Figure 3a). Other patterns of note are the position of other sample members of the gAHB group (Cluster 3) not part of the monophyly. These are mostly WWR samples with an African mitochondrial profile drawn from Texas and likely samples similar to gAHB precursors there (see complementary data for abbreviations) (Figure 3b).

In continental populations, honey bees genetically similar to gAHB could likely be maintained at stable frequencies along the edge of the hybridization zone, often unnoticed or mischaracterized as “EHB” by their behavior and likely to be swept away as AHB keeps expanding. Extirpation to PR and ensuing selection in the island could have preserved these hybrids as their combination of traits was likely adaptive or adapted to oceanic island life. The results of the clustering analysis further reinforce gAHB's position as a population derived from a precursor genotype that is intermediate within the hybridization spectrum of EHB and AHB.

The gAHB population (Cluster 3) placement agrees with the close pattern of the New World clusters that shows a recent and likely ongoing admixture of variable degrees of intensity (see Figure 3a, and Whitfield et al., 2006). In addition, gAHB lies intermediate in the spectrum between EHB and AHB groups (Clusters 8 and 4, Figure 3a).

## 5 CONCLUSION

We conclude that AHB on PR hybridized with EHB and processes of local selection and extraordinary features of the island resulted in an “island bee” currently called gAHB. The ancestral parental gAHB came from Texas. The gAHB population has diverged from its origin (Texas) and is a population with a distinct stable genetic structure. Our results suggest that gAHB may represent a new ecotype of *Apis mellifera*.

## CONFLICT OF INTERESTS

None of the authors have any competing interests.

## AUTHOR CONTRIBUTIONS

JA, AGC, TG, AA: designed research. JA, AGC, TG: performed research. JA, AGC, TG, AA, CWW, DR: contributed new reagents or analytical tools. JA, AGC, TG, AA, DR: analyzed data. JA, AGC, TG, AA, DR, JLUR: wrote the paper.

## Data Availability

Biogeographic origin of samples, SNP Data matrix, and R script Clustering Analyses will available in DRYAD. Provisional https://doi.org/10.5061/dryad.q1857f6
